# The Inhibitory Effect of Flavonoid Aglycones on the Metabolic Activity of CYP3A4 Enzyme

**DOI:** 10.3390/molecules23102553

**Published:** 2018-10-07

**Authors:** Darija Šarić Mustapić, Željko Debeljak, Željan Maleš, Mirza Bojić

**Affiliations:** 1 PDS Biology, Faculty of Science, University of Zagreb, Rooseveltov trg 6, 10000 Zagreb, Croatia; darija.saric.mustapic@halmed.hr; 2 Agency for Medicinal Products and Medical Devices, Ksaverska cesta 4, 10000 Zagreb, Croatia; 3 Institute of Clinical Laboratory Diagnostics, Osijek University Hospital Center, Josipa Huttlera 4, 31000 Osijek, Croatia; debeljak.zeljko@kbo.hr; 4 Department of Pharmacology, School of Medicine, University of Osijek, Cara Hadrijana 10/E, 31000 Osijek, Croatia; 5 Department of Pharmaceutical Botany, Faculty of Pharmacy and Biochemistry, University of Zagreb, Schrottova 39, 10000 Zagreb, Croatia; zmales@pharma.hr; 6 Department of Pharmaceutical Chemistry, Faculty of Pharmacy and Biochemistry, University of Zagreb, A. Kovačića 1, 10000 Zagreb, Croatia

**Keywords:** flavonoids, CYP3A4, HPLC-DAD, testosterone

## Abstract

Flavonoids are natural compounds that have been extensively studied due to their positive effects on human health. There are over 4000 flavonoids found in higher plants and their beneficial effects have been shown in vitro as well as in vivo. However, data on their pharmacokinetics and influence on metabolic enzymes is scarce. The aim of this study was to focus on possible interactions between the 30 most commonly encountered flavonoid aglycones on the metabolic activity of CYP3A4 enzyme. 6β-hydroxylation of testosterone was used as marker reaction of CYP3A4 activity. Generated product was determined by HPLC coupled with diode array detector. Metabolism and time dependence, as well as direct inhibition, were tested to determine if inhibition was reversible and/or irreversible. Out of the 30 flavonoids tested, 7 significantly inhibited CYP3A4, most prominent being acacetin that inhibited 95% of enzyme activity at 1 µM concentration. Apigenin showed reversible inhibition, acacetin, and chrysin showed combined irreversible and reversible inhibition while chrysin dimethylether, isorhamnetin, pinocembrin, and tangeretin showed pure irreversible inhibition. These results alert on possible flavonoid–drug interactions on the level of CYP3A4.

## 1. Introduction

Flavonoids are natural compounds abundantly present in nature, widely distributed in seeds, leaves, and flowers of plants. There are over 4000 flavonoids identified to date, which makes them one of the largest groups of natural products known. They present benzo-γ-pyrone derivatives consisting of phenolic and pyrane rings (Table 1). Based on the difference of the basic structure, flavonoids are classified into several groups: flavans, flavanones, isoflavanones, flavones, isoflavones, anthocyanidines, chalcones, and flavonolignans [1].

It is well-known that the polyphenolic compounds, in particular flavonoids, show various beneficial effects on the human health, that have been demonstrated by various studies. Amongst the many promising health benefits, flavonoids have important biological activities—such as antiallergic, anti-inflammatory, antioxidative, antimicrobial, antitumorigenic, and antimutagenic—thus preventing cancer, heart disease, bone loss, and a number of diseases [2,3,4,5,6,7,8]. Antioxidant property of flavonoids has been most extensively studied and structure-activity (SAR) as well as quantitative structure–activity (QSAR) models have been reported [9,10,11,12,13]. SAR studies showed that modification of flavonoids including hydroxylation and *O*-methylation can influence metabolic faith and their in vitro activity [12].

As many xenobiotics, including flavonoids, are metabolized in vivo, they can interfere with the activity of the metabolic enzymes in the human liver and cause potential risks of drug–herbal drug interactions [14,15,16,17,18,19]. Cytochrome P450 enzymes (CYPs) are the major heme-containing enzyme family and the most important drug-metabolizing enzymes. They play important roles in the metabolism of xenobiotics and the biosynthesis of endogenous molecules (steroids, fatty acids, bile acids, and eicosanoids) [17,20,21]. 75% of the enzymes that metabolize drugs in the human body are cytochrome P450s. Five cytochrome P450s account for 90% of the drug metabolism [22]. The greatest contributor to the biotransformation of drugs is CYP3A4/5 (30.2%), followed by CYP2D6 (20.0%), CYP2C9 (12.8%), CYP1A2 (8.9%), and others [23,24]. Clinically important interactions have been reported between dietary supplements, including functional foods, herbal products, and prescription drugs [25,26,27,28,29,30].

The aim of the present study was to evaluate the inhibitory effect of thirty flavonoid aglycones, most commonly found in herbal drugs and propolis, on the most important drug metabolizing enzyme CYP3A4. Although flavonoids are found mainly as glycosides in natural sources, they are susceptible to hydrolysis in gut [31]. Thus, they reach systemic circulation as aglycones, and as such are exposed to the cytochrome P450 enzymes. The selection of flavonoid aglycones was arbitrary, and reflects most commonly found aglycones in propolis and herbal medicinal drugs of Lamiaceae family found in Croatia [1].

For enzyme activity assessment testosterone 6β-hydroxylation was used as it represents a marker reaction of CYP3A4 activity. Generated product/metabolite was monitored by high performance liquid chromatography coupled with diode array detector (HPLC-DAD). If the inhibitory effect has been observed, the type of inhibition was determined as this can have repercussion on the possible drug–flavonoid interactions.

## 2. Results and Discussion

**Figure 1:**
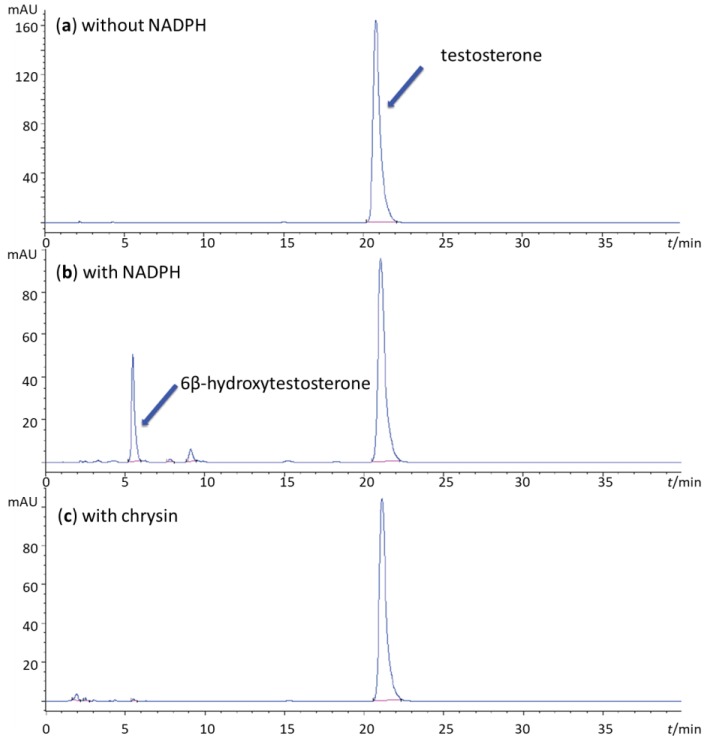
Chromatogram of testosterone oxidation by recombinant baculovirus system with hyper expressed CYP3A4, monitored at 240 nm. (**a**) Incubation without the NADPH-generating system. The testosterone substrate is shown at *t*_R_ = 20.7 min. (**b**) Incubation with the NADPH generating system, showing the metabolite, 6β-hydroxytestosterone, at *t*_R_ = 5.4 min. (**c**) Incubation with the NADPH-generating system and flavonoid (chrysin). Significant inhibition of the CYP3A4 activity is observed as the production of the major testosterone metabolite is significantly lower (*t*_R_ = 5.4 min).

To test the CYP3A4 enzyme inhibition, residual activity was assessed based on the product of the marker reaction generated by monitoring incubations with addition of flavonoid, as a potential inhibitor, vs. incubations without inhibitor. Figure 1 shows an example of the typical inhibitor, chrysin. Positive control (incubation with substrate, and the NADPH generating system) shows production of 6β-hydroxytestosterone as major metabolite of testosterone hydroxylation. Negative control (incubation with substrate and without the NADPH generating system) shows no product generation, confirming that 6β-hydroxytestosterone is product of generated by CYP3A4 enzyme. When chrysin is added to the incubation mixture (containing substrate, and the NADPH generating system), 6β-hydroxytestosterone production is depleted. Troleandomycin, an irreversible inhibitor of CYP3A4, used as a positive control in 25 µM concentration caused complete inhibition of CYP3A4, while 0.15 µM ketoconazole, a direct reversible inhibitor of CYP3A4, reduced enzyme activity by 80 ± 4% (*p* < 0.001).

Screening of all flavonoids was conducted against metabolism dependent inhibition experimental setup, as it includes preincubation required for time dependent inhibition, as well as incubation of flavonoids and marker substrate at the same time, consequently detecting direct inhibition. Seven flavonoids have been shown to inhibit CYP3A4 with statistical significance *p* < 0.05.

Flavanones acacetin and pinocembrin decreased enzyme activity by 95% and 50% respectively, acacetin being the most prominent inhibitor (Table 1). Acacetin has positive effects cardiomyocyte protection, antibacterial activity as well as anticancer properties [32,33,34]. Doostdar et al. studied the influence of acacetin on CYP1 family and reported inhibitory effect on CYP1A subfamily and CYP1B1 enzyme [35]. However, there are no reports on CYP3A4 inhibition.

Pinocembrin has been extensively studied due to its positive effects on cardiovascular system. This flavanone protects endothelial cells from oxidized LDL-induced injury, and has neuroprotective effects on ischemia/reperfusion-induced brain injury by inhibiting autophagy [36,37]. Although pharmacokinetic of pinocembrin was studied, no CYP3A4 inhibition was reported [38]. In this study, pinocembrin decreased enzyme activity by 50% (Table 1).

Residual activity of CYP3A4 after incubations with flavones apigenin, chrysin, chrysin dimethyl ether, and tangerine was 24%, 17%, 61%, and 42% (Table 1). Apigenin has been well studied due to it positive effects in prevention of breast cancer, and positive effects on cardio myoblasts and pancreatic cells in pancreatitis [39,40,41,42,43]. At 1 µM concentration, decrease in activity of 76% for was observed apigenin. Reversible inhibition of CYP3A4 by apigenin was reported by Li et al. with an inhibition constant of 6.2 µM, while Kimura et al. reported mix type inhibition [44,45]. Both these studies indicate importance of reversible inhibition. However, based on our study metabolism dependent inhibition of CYP3A4 by apigenin, here reported for the first time, is of greater importance.

Chrysin dimethylether has been reported to have antitrypanosomal and antileishmanial properties while chrysin inhibits diabetic renal tubulointerstitial fibrosis, xanthine oxidase activity, and foam cell formation having potentially positive effects in diabetes, gout, and atherosclerosis [46,47,48,49]. Neither chrysin, nor chrysin dimethylether were reported as inhibitors of CYP3A4 previously. Although the effect of chrysin on 1′-hydroxilation of midazolam (another marker reaction of CYP3A4 enzyme) was studied, inhibition of CYP3A4 activity was not observed [50].

Tangeretin, a component of orange juice, has been reported to have positive effects on human health that include antioxidant, antiviral, and anticancer properties [51,52,53]. Quintieri et al. reported that the nonsubstituted flavone and the pentamethoxy-substituted tangeretin, lacking free hydroxyl groups in their A and B rings, stimulate, rather than inhibit, the metabolism of midazolam [50]. However, in our study in which residual activity of CYP3A4 was studied on testosterone as marker substrate, an inhibitory effect was observed. Our results are in accordance with Obermeier et al. who reported that tangeretin inhibited CYP3A4 mediated nifedipine oxidation in human liver microsomes in an uncompetitive manner with inhibition constant of 72 µM [54]. Lastly, Takanaga et al. reported no influence of tangeretin, neither activation, nor inhibition, on CYP3A4 activity when using testosterone as marker substrate on human liver microsomes and recombinant CYP3A4 systems [55]. Observed difference justifies recommendations of medicinal products regulatory agencies to use at least two marker substrates when studding CYP3A4 activity [56].

Isorhamnetin was the only flavonol to show inhibitory effect decreasing residual enzyme activity to 73% (Table 1). Isorhamnetin has lately been extensively studied as supportive agent in therapy of non-small cell lung carcinoma when combined with cisplatin and carboplatin by enhancing anticancer properties of platina derivatives and apoptosis [57]. On animal models, it has been also shown acts against influenza as well as attenuates collagen induced arthritis [58,59]. Ding et al. did not observe change in activity of CYP3A4 treated with isorhamnetin on hepatocyte carcinoma cell line HepG2 [60]. Ekstrand et al. [61] used a porcine animal model to assess the influence of isorhamnetin depending on the gender. Inhibition was assessed to be competitive with inhibition constants of 71 and 94 µM for male and female pigs, respectively. Under 16 µM concentration no competitive inhibition was observed, which is in accordance with our results [61]. However, under 1 µM concentration of isorhamnetin, we have observed metabolism dependent inhibition that has not been previously reported.

Interestingly, stimulation of enzyme activity of CYP3A4 was observed with tamarixetin; observed residual activity was 195% (Table 1). Tamarixetin exhibits anti-inflammatory activity, and prevents bacterial sepsis by stimulating immune system and interleukin 10 production [62]. It also has anticancer properties on human leukemia cells [63]. Li et al. have shown that tamarixetin has no influence on CYP3A4 induction through pregnane X receptor, constitutive androstane receptor, and aryl hydrocarbon receptor [64]. Our observation of stimulation of CYP3A4 activity by tamarixetin is in contrast to von Moltke et al. who observed 50% inhibition at sub 10 µM concentration [65]. Although stimulation in vitro (not mediated by increased induction of enzyme in vivo) is rarely observed, it has been reported that anticancer drug gefitinib is a potent stimulator of CYP3A4 [66].

**Figure 2:**
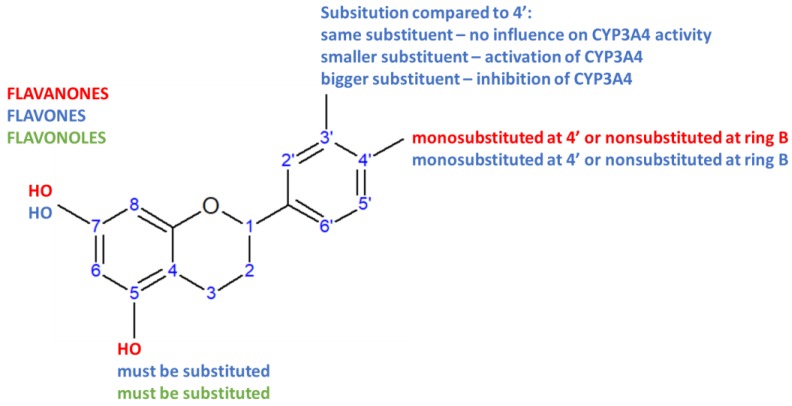
Contribution of structural features to the inhibitory effect (if not otherwise stated) of different classes of flavonoids on the activity of CYP3A4 enzyme. Each class is represented with a different color.

Looking at the structural features responsible for the inhibitory effect (Table 1, Figure 2), it can be observed that flavanones and flavones hydroxylated at the positions 5 and 7 of the A ring, and monosubstituted at the position 4′ of the B ring, decrease CYP3A4 enzyme activity regardless of group present (methoxy or hydroxyl) e.g., acacetin and apigenin. As the hydroxyl group at the B ring is nonionizable under the physiological pH, hydrophobic interactions of the flavonoid with the CYP3A4 are probably of importance. In case of flavanones and flavonoles, the B ring does not necessarily need to be substituted for the inhibitory effect to be observed e.g., pinocembrin, chrysin, chrysin-dimethylether. However, in that instance hydroxyl group should not be methylated, and susceptible to ionization under physiological conditions, indicating importance of ion–ion interactions between flavanones and CYP3A4 enzyme. At the same time, flavanones and flavonoles have to be substituted at the position 5 for the inhibitory effect to be observed. In case of flavonoles, it is interesting to observe that disubstituted flavonoids at the positions 3′ and 4′ of the B ring do not influence enzyme activity if both substituents are hydroxyl groups (quercetin and rhamnetin), increase enzyme activity if substituent at the position 4′ is bigger in size than substituent at the position 3′ (tamarixetin) while inhibition is observed when substituent at the position 3′ is greater than the one at the position 4′ (isorhamnetin).

To distinguish if the observed metabolism dependent inhibition (of the seven confirmed inhibitors of CYP3A4 in this study) was a result of time dependent inhibition, in which additional time is needed for the inhibitor to interact with enzyme to cause the inhibition, preincubations were conducted without presence of NADPH coenzyme. In this way, no reduction of CYP3A4 heme iron to Fe^2+^ form occurred, giving sufficient time for the potential inhibitor to interact with the enzyme.

**Figure 3:**
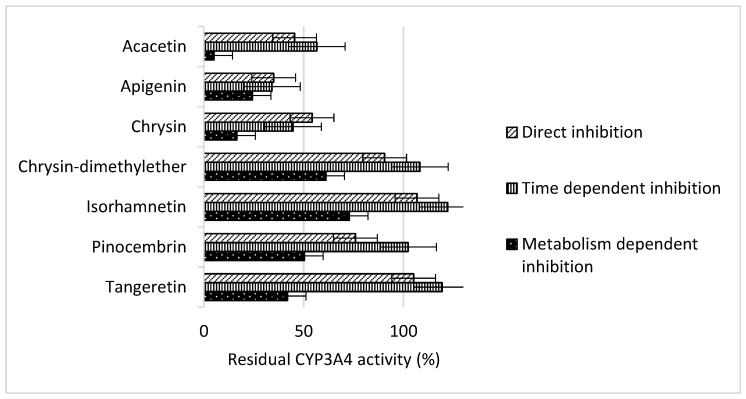
Residual enzyme activity of CYP3A4 determined after incubation with individual flavonoids, and expressed as a percentage of control reaction without addition of flavonoids. Flavonoids were preincubated with enzyme with and without NADPH coenzyme to test metabolic, and time dependent inhibition, respectively. Direct inhibition had no preincubation.

In time dependent inhibition assay, inhibition was observed with acacetin, apigenin, and chrysin. Residual CYP3A4 activity was 57 ± 10% (*p* = 0.047), 34 ± 10% (*p* = 0.005), and 45 ± 3% (*p* = 0.019), respectively (Figure 3). However, no statistical difference was observed in residual activity of CYP3A4 enzyme for apigenin between metabolism and time dependent inhibition assays, indicating that apigenin is pure reversible inhibitor of CYP3A4. No time dependent inhibition was observed with chrysin dimethylether, isorhamnetin, pinocembrin, and tangeretin, and this was confirmed in direct inhibition assay, indicating these four flavonoids are irreversible inhibitors of CYP3A4.

In direct inhibition assay no preincubation is conducted, and this experimental setup is designed to detect reversible inhibition in which substrate and inhibitor compete at the same time for the active site. Direct inhibition assay showed inhibitory effect of acacetin, apigenin, and chrysin on CYP3A4 by decreasing residual enzyme activity to 46 ± 6% (*p* = 0.005), 35 ± 18% (*p* = 0.049), and 54 ± 23% (*p* = 0.044), respectively (Figure 2). These results show that no real time dependent inhibition is present, and observed inhibition of CYP3A4 in time dependent inhibition assay is consequence of the direct inhibition.

Time dependent inhibition is extremely rare for human cytochromes P450 and has been previously reported only for cilengitide, an experimental anticancer drug used for treatment of glioblastoma, and CYP3A4 enzyme in which inhibitor was in extremely high millimolar concentration [67].

Direct inhibition is reversible and its clinical implications, i.e., drug–drug interactions, can be avoided if inhibitor is discontinued from the treatment and substitute with another drug. More important interactions are those irreversible, that have been demonstrated for six flavonoids acacetin and chrysin (in combination with reversible inhibition), and chrysin dimethylether, isorhamnetin, pinocembrin, and tangeretin (pure irreversible inhibitors). Irreversible inhibition cannot be overcome by discontinuation and substitution of a medicinal drug, as the enzyme is inactivated and time is needed for a new enzyme to be re-expressed. As the half-life of cytochromes P450 is between 24 and 72 h, regeneration of enzyme activity can take up to two weeks, preventing successful therapy. This is especially of importance in cases where medicinal drug is exclusively metabolized by inhibited enzyme or inhibited enzyme is susceptible to genetic polymorphism, as well as concomitant use of drugs and flavonoid rich foods metabolizing through same enzyme due to possible drug–flavonoid interactions [56].

A significant number of papers on metabolism of flavonoids and their inhibition of metabolic enzymes has been published [44,45,50,54,55,56,61,64,65]. However, as this class of compounds covers thousands of substances, metabolic data and inhibitory effects on the majority of them is still missing. Moreover, data published is not always confirmed by subsequent studies, e.g., tamarixetin is activator of CYP3A4 in our study, while previously it has been reported that it either has no effect or it inhibits CYP3A4 [64,65]. For the first time we have reported inhibition of CYP3A4 by acacetin, pinocembrin, chrysin, and chrysin dimethylether, and we have also shown that pinocembrin and isorhamnetin act as irreversible inhibitors of CYP3A4. Catalytic cycle of cytochromes P450 can be affected by inhibitor at different stages [56]. Reversible inhibitors usually affect first step of the catalytic cycle, i.e., binding of the substrate in case of most commonly observed competitive inhibition [56]. Irreversible inhibitors usually affect cytochromes P450 when radical form of substrate is generated forming a reactive intermediate product that can covalently bind to the enzyme and cause inactivation [56]. Sometimes inhibitors can have both reversible and irreversible effect on the enzyme as it has been shown with cilengitide and CYP3A4 [67]. Further studies of chrysin dimethylether, isorhamnetin, pinocembrin, and tangeretin inhibition of CYP3A4 are needed to determine inactivation kinetics and steps of the catalytic cycle of cytochrome P450 3A4 that are affected by the inhibitor or potential reactive intermediate products/metabolites.

## 3. Materials and Methods

### 3.1. Materials

Thirty flavonoids used in this study (3,6-dihydroxyflavon, 3,7-dihydroxyflavon, 6-hydroxyflavon, 7-hydroxyflavon, acacetin, apigenin, catechin, chrysin, chrysin dimethyleter, diosmetin, flavanone, flavone, galangin, genistein, hesperetin, isorhamnetin, kaempferol, luteolin, morin, myricetin, naringenin, pinocembrin, pinocembrin-7-metyleter, prunetin, quercetin, quercetin, rhamnetin, sakuranetin, tamarixetin, and tangeretin) were acquired commercially from Sigma-Aldrich (St. Louis, MO, USA), TransMIT GmbH (Gießen, Germany), Extrasynthèse (Genay, France). For the evaluation of activity of CYP3A4 testosterone was used as marker substrate (Sigma-Aldrich). 6β-hydroxytestosterone was purchased from Cayman Europe (Tallinn, Estonia). Positive controls used to test direct inhibition (ketoconazole) and irreversible inhibition (troleandomycin) were obtained from Sigma-Aldrich (St. Louis, MO, USA).

The recombinant baculosomes with hyperexpressed CYP3A4 and coexpressed NADPH cytochrome P450 reductase and cytochrome b_5_, was used as source of enzyme. Baculosomes were commercially obtained from Thermo Fisher Scientific (Waltham, MA, USA), and all other chemicals from Sigma-Aldrich if not otherwise indicated. A 1.0 M solution phosphate buffer of pH 7.4 was prepared in house. NADPH generating system was prepared of 0.1 M glucose-6-phosphate, 10 mg mL^−1^ NADP^+^, and 1000 IU mL^−1^ glucose-6-phosphate dehydrogenase in ratio 100:50:2 (*v*/*v*/*v*) [67].

### 3.2. CYP3A4 Inhibition Assays

6β-Hydroxylation of testosterone was used as marker reaction to monitor activity of the CYP3A4 enzyme [67]. Incubations were conducted at 37 °C in the total volume of 100 µL. pH was set to 7.4 using the potassium phosphate buffer (final concentration 50 mM). Incubation mixture contained 5 pmol of the CYP3A4 enzyme. Residual activity was determined by coincubation flavonoid (1 µM) with a marker substrate (200 µM testosterone) for 30 min, with or without preincubation depending of inhibition type tested. Reactions were initiated by adding 15 µL of NADPH generating system. Each reaction mixture was quenched by the addition of four volumes of dichloromethane and centrifuged at 1900× *g* for 10 min. The organic layer was transferred, and the solvent was evaporated under a stream of nitrogen. The dried sample was dissolved in methanol for HPLC analysis [67].

### 3.3. Determination of the Inhibition Type

Three types of experiments were conducted to determine metabolism dependent inhibition, time dependent inhibition, and direct inhibition [67]. To determine metabolism dependent inhibition, flavonoids were first preincubated with enzymes with the addition of the generating system for 30 min, after which testosterone was added to determine residual activity. Incubation reaction was quenched after 15 min by adding organic solvent.

If metabolism-dependent inhibition was determined, time dependent inhibition was assessed by preincubating flavonoids and enzymes, after which residual activity was determined by adding NADPH generating system along with substrate. In this instance, the direct inhibition was also tested without preincubation, i.e., NADPH generating system, was added to incubation mixture containing flavonoid and substrate [67].

### 3.4. HPLC-DAD Analysis

HPLC analysis was performed on Agilent 1100 instrument (Santa Clara, CA, USA) coupled with diode array detector on Agilent Zorbax SB C18 column (4.6 × 250 mm, 3 μm). The mobile phase used consisted of 64% methanol and 36% water; analysis was isocratic at a flow rate of 1.0 mL min^−1^ Chromatograms were recorded at 240 nm, and the amount of generated product (6β-hydroxytestosterone) was determined as the area under the curve based on the calibration curve of the standard [67].

### 3.5. Statistical Analysis

All incubations were conducted in triplicate. The results are expressed as residual activity of the enzyme, i.e., percentage of product generated in incubation with the addition of flavonoid in ratio to the control without flavonoid. Statistical significance was tested with Student’s *t*-test in the program R (The R Project for Statistical Computing, Vienna, Austria). Mann–Whitney U-test was used to test normality (*p* = 0.508) and Levene’s test homogeneity (*p* = 0.882) of the inhibitory data for apigenin justifying the use of the selected statistical analysis.

## 4. Conclusions

Out of 30 flavonoids screened for potential CYP3A4 enzyme inhibition, 7 showed statistically significant inhibition. Apigenin showed reversible inhibition; acacetin and chrysin showed combined irreversible and reversible inhibition; while chrysin dimethylether, isorhamnetin, pinocembrin, and tangeretin showed pure irreversible inhibition. These results bring attention to possible flavonoid–drug interactions on the level of CYP3A4. As CYP3A4 is the most significant enzyme for the metabolism of xenobiotics including drugs, further in vivo studies are needed to assess clinical significance of the observed inhibitions, and possible drug–flavonoid interactions.

## Figures and Tables

**Table 1:** Structural characteristics of flavonoids screened as potential inhibitors of CYP3A4 enzyme. Results of screening of metabolism dependent inhibition are expressed as residual enzyme activity when compared to the control without the addition of flavonoid. *p* denotes statistical significance.

Basic Skeleton of Flavonoids				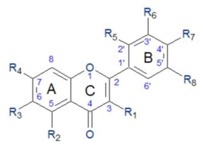							
	Flavonoid	R_1_	R_2_	R_3_	R_4_	R_5_	R_6_	R_7_	R_8_	Residual Activity (%)	*p*
	**Flavanones**										
1	Acacetin	H	OH	H	OH	H	H	OCH_3_	H	5 ± 4	0.007
2	Flavanone	H	H	H	H	H	H	H	H	86 ± 46	0.361
3	Hesperetin	H	OH	H	OH	H	H	OCH_3_	OH	49 ± 19	0.065
4	Pinocembrin-7-methylether	H	OH	H	OCH_3_	H	H	H	H	83 ± 2	0.147
5	Pinocembrin	H	OH	H	OH	H	H	H	H	50 ± 15	0.003
6	Sakuranetin	H	OH	H	OCH_3_	H	H	OH	H	94 ± 45	0.439
	**Flavones**										
7	6-hydroxyflavone	H	H	OH	H	H	H	H	H	83 ± 14	0.220
8	7-hydroxyflavone	H	H	H	OH	H	H	H	H	78 ± 14	0.172
9	Apigenin	H	OH	H	OH	H	H	OH	H	24 ± 3	0.013
10	Chrysin	H	OH	H	OH	H	H	H	H	17 ± 3	0.010
11	Chrysin-dimethylether	H	OCH_3_	H	OCH_3_	H	H	H	H	61 ± 21	0.049
12	Diosmetin	H	OH	H	OH	H	OH	OCH_3_	H	172 ± 82	0.169
13	Flavone	H	H	H	H	H	H	H	H	81 ± 16	0.087
14	Luteolin	H	OH	H	OH	H	OH	OH	H	112 ± 31	0.356
15	Naringenin	H	OH	H	OH	H	H	OH	H	65 ± 24	0.155
16	Tangeretin ^1^	H	OCH_3_	OCH_3_	OCH_3_	H	H	OCH_3_	H	42 ± 3	0.027
17	Techtocrysin	H	OH	H	OCH_3_	H	H	H	H	102 ± 15	0.449
	**Flavonoles**										
18	3,6-dihydroxyflav.	OH	H	OH	H	H	H	H	H	100 ± 14	0.220
19	3,7-dihydroxyflav.	OH	H	H	OH	H	H	H	H	91 ± 27	0.375
20	Galangin	OH	OH	H	OH	H	H	H	H	48 ± 24	0.093
21	Isohramnetin	OH	OH	H	OH	H	OCH_3_	OH	H	73 ± 6	0.048
22	Kaempferol	OH	OH	H	OH	H	H	OH	H	101 ± 14	0.449
23	Morin	OH	OH	H	OH	OH	H	OH	H	122 ± 8	0.061
24	Myricetin	OH	OH	H	OH	H	OH	OH	OH	133 ± 35	0.195
25	Quercetin	OH	OH	H	OH	H	OH	OH	H	126 ± 10	0.152
26	Rhamnetin	OH	OH	H	OCH_3_	H	OH	OH	H	117 ± 84	0.386
27	Tamarixetin	OH	OH	H	OH	H	OH	OCH_3_	H	195 ± 29	0.023
	**Isoflavones**										
28	Genistein	H	OH	H	OH	H	H	OH	H	72 ± 24	0.179
29	Prunetin	H	OH	H	OCH_3_	H	H	OH	H	74 ± 14	0.149
	**Catechins** ^2^										
30	Catechin	OH	OH	H	OH	H	H	OH	OH	98 ± 10	0.441

^1^ Additional methoxy group at the position 8. ^2^ No keto group at the position 4.
